# The gut microbiome and gastrointestinal cancers: mechanisms, biomarkers and therapeutic opportunities

**DOI:** 10.3389/fphys.2025.1676796

**Published:** 2025-10-30

**Authors:** Sarbjeet Kaur Makkar, Kumar S. Bishnupuri

**Affiliations:** ^1^ Department of Internal Medicine, Division of Hematology and Oncology, University of Michigan, Ann Arbor, MI, United States; ^2^ Rogel Cancer Center, University of Michigan, Ann Arbor, MI, United States; ^3^ Division of Gastroenterology, Department of Medicine, Washington University School of Medicine, St. Louis, MO, United States

**Keywords:** gut microbiome, gastrointestinal cancers, microbial dysbiosis, cancerimmunotherapy, microbiome-derived biomarkers, microbiota-targeted therapy

## Abstract

Gastrointestinal (GI) cancers remain a leading global cause of cancer-related mortality, significantly impacting public health and healthcare systems worldwide. Emerging evidence underscores the critical role of gut microbiome dysbiosis—characterized by disrupted microbial diversity and function—in GI carcinogenesis. Utilizing recent advancements in multi-omics technologies and sophisticated computational biology, researchers have elucidated distinct microbial signatures associated with colorectal, gastric, hepatobiliary, pancreatic, and esophageal cancers. This review comprehensively analyzes the primary mechanisms through which gut microbes contribute to cancer development and progression, encompassing genotoxicity, chronic inflammation, metabolic dysregulation, epigenetic modifications, and immunomodulation. Moreover, we explore innovative microbiome-derived biomarkers for potential clinical applications, including early diagnosis, prognosis assessment, and therapeutic response prediction. The intricate interactions between microbiota and standard cancer therapies—chemotherapy, immunotherapy, and radiation therapy—are discussed, highlighting microbiome influences on therapeutic efficacy and adverse effect profiles. We also critically assess the impact of modifiable factors such as diet, medications, lifestyle, and environmental exposures on microbiome composition and cancer risk. The review evaluates emerging therapeutic interventions, including dietary modifications, probiotics, prebiotics, fecal microbiota transplantation (FMT), and engineered live biotherapeutics. Despite notable advancements, significant hurdles remain, including clarifying causality, methodological standardization, and equitable global research representation. Addressing these challenges, we propose a strategic research agenda aimed at harnessing microbiome insights to advance precision oncology and improve GI cancer outcomes globally.

## 1 Introduction

Gastrointestinal (GI) cancers—comprising colorectal, gastric, liver, pancreatic, biliary, and esophageal malignancies—pose an escalating global health threat, accounting for over 4.4 million deaths annually (Sung et al., 2021). This rising incidence, projected to increase by approximately 58% by 2040, is influenced by demographic transitions, increased obesity rates, dietary shifts toward processed foods, and reduced physical activity (Arnold et al., 2020; Bray et al., 2018). Despite substantial progress in diagnosis and treatment modalities, the prognosis for many GI cancers remains unfavorable, notably for pancreatic (<12%) and hepatobiliary cancers (<20%) (Ferlay et al., 2019).

Historically, the recognition of microbial contributions to GI cancers began with the identification of *Helicobacter pylori* as a gastric carcinogen in 1984 (Marshall and Warren, 1984). Subsequent advances in sequencing technologies expanded this paradigm, revealing complex microbial communities—collectively termed microbiomes—as integral regulators of tumor initiation, progression, and response to treatments (Garrett, 2015). These microbial ecosystems interact bidirectionally with host genetics, immune function, metabolism, and environmental factors, profoundly influencing cancer pathophysiology (Schwabe and Jobin, 2013; Brennan and Garrett, 2016).

Technological innovations have significantly accelerated microbiome research, enabling precise microbial profiling through metagenomics, metatranscriptomics, metaproteomics, and metabolomics (Qin et al., 2010; Proctor, 2019). Complementing these advances, computational methodologies, including machine learning, network analysis, and causal inference frameworks, have transformed descriptive microbial datasets into mechanistic understanding (Zeller et al., 2014; Chen et al., 2010). Furthermore, novel spatial-omics and single-cell analytical techniques are now elucidating detailed microbe-host interactions within tumor microenvironments, providing unprecedented spatial resolution (Shi et al., 2022; Geva-Zatorsky et al., 2017).

This review integrates current knowledge, systematically discussing microbial dysbiosis across specific GI cancers, elucidating mechanistic pathways, evaluating microbiome-based biomarkers, and examining interactions between microbiota and cancer therapies. It also highlights how diet, medications, lifestyle, and environmental exposures modulate microbial communities, and critically appraises microbiome-targeted therapeutic interventions. In this narrative review, we synthesize current evidence placing the gut microbiome at the intersection of gastrointestinal (GI) cancer biology and precision oncology. Drawing on findings from epidemiological studies, mechanistic research, and clinical trials, we provide an integrated perspective on how the microbiome influences GI cancers. We first describe disease-specific dysbiosis patterns, then dissect key mechanistic pathways linking microbial activity to tumor development and progression We further evaluate emerging microbial biomarkers for diagnosis and prognosis and examine the interplay between the microbiome and cancer therapies. Environmental modulators of the microbiome and therapeutic strategies targeting the microbiome are also discussed. We conclude by identifying current knowledge gaps and outlining future directions for research in this rapidly evolving field.

## 2 Microbiome dysbiosis across major GI cancers

### 2.1 Colorectal cancers

Exhibit distinct microbial signatures characterized by enrichment of pathogenic bacteria such as *Fusobacterium* nucleatum, *Escherichia coli* strains possessing the polyketide synthase (pks) genomic island, and enterotoxigenic *Bacteroides fragilis*. These pathogens are consistently associated with colorectal carcinogenesis and poor patient outcomes (Sears, 2009; Tilg and Adolph, 2015; Kostic et al., 2013). In particular, *Fusobacterium* nucleatum promotes tumorigenesis by modulating immune responses, facilitating cellular proliferation, and influencing chemotherapy resistance, while pks + *E. coli* strains produce genotoxic colibactin, directly inducing DNA damage and mutagenesis (Cuevas-Ramos et al., 2010; Nougayrède et al., 2006).

### 2.2 Gastric cancer

Gastric Cancer is associated with a shift in microbiome composition toward increased abundance of *Streptococcus*, Prevotella, and nitrosating bacterial species capable of generating carcinogenic N-nitroso compounds. Persistent dysbiosis following *Helicobacter pylori* eradication strongly correlates with increased risk of progression to intestinal-type adenocarcinoma, underscoring the role of the broader microbiome rather than a single pathogen in gastric carcinogenesis (Yu et al., 2024; Plummer et al., 2015).

### 2.3 Esophageal cancer

In Barrett’s esophagus and esophageal adenocarcinoma, microbiome dysbiosis predominantly involves increased colonization by gram-negative anaerobes, which metabolize bile acids and exacerbate inflammation through interleukin-8 (IL-8) mediated pathways (Sharma et al., 2022; Blackett et al., 2022). Notably, Porphyromonas-positive squamous cell carcinoma tumors exhibit heightened PD-L1 expression, implicating these microbes in immune modulation and potentially influencing responsiveness to immunotherapies (Yagi et al., 2019).

### 2.4 Pancreatic cancer

Pancreatic tumors are characterized by distinct microbial communities, notably enriched in Proteobacteria, Enterobacteriaceae, Malassezia, and *Fusobacterium* species. Evidence indicates that microbial translocation from the gut to the pancreas can activate innate immune pathways and potentiate inflammation-driven carcinogenesis. Such intratumoral microbial colonization may contribute significantly to tumor progression and resistance to chemotherapy, thus highlighting the potential for microbiome-targeted therapeutic strategies in pancreatic cancer management (Pushalkar et al., 2018; Riquelme et al., 2019; Ren et al., 2017).

### 2.5 Hepatobiliary cancers

Hepatobiliary malignancies exhibit characteristic microbial alterations linked closely to chronic liver disease. Gut microbiota dysbiosis, notably in the context of cirrhosis, promotes hepatic inflammation and fibrosis primarily through Toll-like receptor 4 (TLR4) mediated signaling pathways (Seki et al., 2007; Schnabl and Brenner, 2014). Furthermore, enrichment of Akkermansia muciniphila has been associated with improved responses to immune checkpoint inhibitors in hepatocellular carcinoma, providing a predictive biomarker and potential therapeutic target (Zheng et al., 2019). Additionally, shifts in biliary microbiota have been correlated with the development of cholangiocarcinoma, emphasizing the importance of microbiome monitoring in hepatobiliary oncology (Ridlon et al., 2014).

## 3 Mechanistic pathways linking the gut microbiome to GI tumorigenesis

**FIGURE 1:**
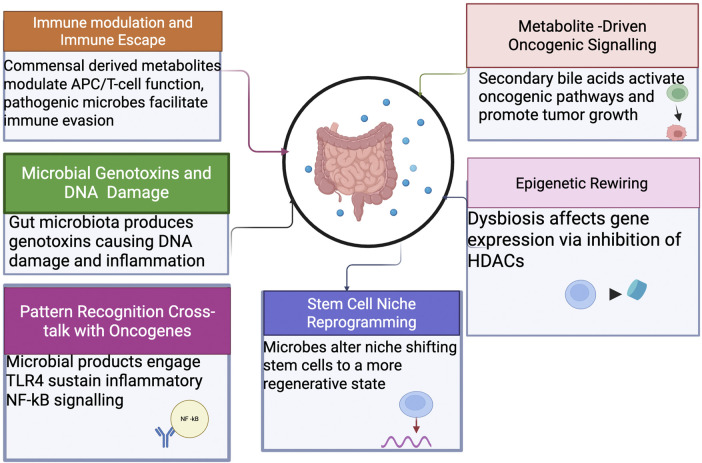
Mechanistic pathways linking the gut microbiome to GI tumorigenesis. The microbiome promotes tumor initiation and progression through immune modulation, genotoxin production (e.g., colibactin, BFT), metabolite-driven oncogenic signaling (secondary bile acids), chronic inflammation via pattern-recognition receptors, epigenetic alterations, and stem cell niche reprogramming.

The gut microbiome plays a pivotal role in gastrointestinal (GI) tumorigenesis through a multifaceted network of mechanisms. These include modulation of immune responses, induction of DNA damage, alteration of host metabolism, engagement with pattern recognition receptors, epigenetic modifications, and reprogramming of the stem cell niche. Understanding these pathways provides insights into potential targets for prevention and therapy (Figure 1).

### 3.1 Immune modulation and immune escape

The gut microbiome significantly influences immune homeostasis and tumor immune surveillance. Commensal-derived metabolites, including short-chain fatty acids (SCFAs), modulate antigen-presenting cell function and T-cell differentiation, promoting anti-inflammatory and anti-tumor responses (Belkaid and Hand, 2014). Conversely, pathogenic microbes like *Fusobacterium* nucleatum employ mechanisms such as the Fap2 protein, which engages inhibitory receptors (e.g., TIGIT) on immune cells, creating immune-privileged tumor microenvironments and facilitating tumor immune evasion (Gur et al., 2015).

### 3.2 Microbial genotoxins and DNA damage

Certain gut microbiota species produce genotoxins such as colibactin (pks + *Escherichia coli*) and *Bacteroides fragilis* toxin (BFT). Colibactin induces specific DNA alkylation damage associated with a unique mutational signature (COSMIC SBS 88), significantly contributing to colorectal carcinogenesis (Nougayrède et al., 2006). BFT promotes epithelial disruption and β-catenin activation, potentiating chronic inflammation and tumorigenesis via IL-17-driven pathways (Wu et al., 2003).

### 3.3 Metabolite-driven oncogenic signaling

Microbial metabolites, notably secondary bile acids like deoxycholic and lithocholic acids, are implicated in cancer progression. These metabolites can activate oncogenic signaling pathways such as FXR–SHP and YAP/TAZ, leading to increased cellular proliferation and tumor growth, especially in hepatocellular carcinoma (Yoshimoto et al., 2013). Reduced abundance of beneficial microbes producing SCFAs exacerbates oxidative stress and metabolic dysregulation in colonocytes, favoring pro-tumorigenic environment (Louis et al., 2014).

### 3.4 Pattern recognition

Cross-talk with Oncogenes: Microbial products such as lipopolysaccharide (LPS) engage host pattern recognition receptors, particularly TLR4, leading to sustained inflammatory signaling via NF-κB pathways. This inflammatory environment synergizes with host genetic mutations (e.g., KRAS, TP53) to drive tumor initiation and progression (Rakoff-Nahoum et al., 2004).

### 3.5 Epigenetic rewiring

Gut microbiome dysbiosis influences host epigenetic modifications, notably through butyrate-mediated inhibition of histone deacetylases (HDACs). Butyrate-mediated HDAC inhibition leads to hyperacetylation of histones, altering gene expression patterns involved in tumor suppression and inflammation (Donohoe et al., 2012). Conversely, reduced butyrate levels due to dysbiosis contribute to DNA hypermethylation of tumor suppressor genes, accelerating carcinogenesis (Louis et al., 2014).

### 3.6 Stem cell niche reprogramming

Microbial interactions alter stem cell dynamics, promoting tumor initiation and progression. Enterotoxigenic *B. fragilis* and high-fat dietary patterns shift intestinal stem cells toward a regenerative, foetal-like phenotype marked by enhanced plasticity, significantly increasing susceptibility to tumorigenic mutations and promoting tumorigenesis (Schell et al., 2020).

## 4 Microbiome-derived biomarkers for early detection, prognosis, and treatment selection

### 4.1 Non-invasive early detection tools

Traditional screening methods for colorectal cancer (CRC), such as fecal immunochemical tests (FIT), have limitations in sensitivity and specificity. Recent studies have demonstrated that metagenomic profiling of fecal samples can identify distinct microbial signatures associated with early-stage CRC. For instance, Yu et al. (2017) reported that specific microbial markers could distinguish CRC patients from healthy controls with high accuracy, suggesting their potential as non-invasive diagnostic tools. Similarly, Zeller et al. (2014) found that integrating microbial biomarkers with FIT improved the detection rate of CRC, particularly in early stages.

### 4.2 Risk stratification and surveillance: integrating microbiome and host genetics

Beyond detection, the gut microbiome holds promise in stratifying individuals based on cancer risk. Polygenic microbiome risk scores, which combine host genetic factors with microbial composition data, have been developed to predict the likelihood of adenoma progression and CRC development (Vogtmann et al., 2016). Moreover, salivary microbiome profiling has emerged as a potential tool for identifying precancerous conditions. Chen et al. (2015) demonstrated that specific ratios of oral bacteria, such as Porphyromonas endodontalis to Prevotella melaninogenica, were predictive of intestinal metaplasia in gastric cancer, highlighting the utility of oral microbiota as biomarkers for early intervention.

### 4.3 Predictive biomarkers of therapy response: microbiome’s role in treatment efficacy

The composition of the gut microbiome significantly influences the efficacy of cancer therapies. High intratumoral levels of *Fusobacterium nucleatum* have been associated with reduced responsiveness to adjuvant chemotherapy in CRC patients. Mima et al. (2016) found that patients with elevated *F. nucleatum* levels had shorter survival times, suggesting that microbial profiling could inform treatment decisions. Conversely, beneficial microbes like Akkermansia muciniphila and members of the Ruminococcaceae family have been linked to favorable responses to immune checkpoint inhibitors. Studies by Gopalakrishnan et al. (2018) and Routy et al. (2018) indicated that the presence of these bacteria correlated with improved outcomes in patients undergoing immunotherapy, underscoring the potential of microbiome modulation to enhance treatment efficacy.

## 5 Microbiome interplay with cancer therapies and supportive care

The gut microbiome significantly modulates cancer treatment efficacy and toxicity through its influence on chemotherapy, immunotherapy, and radiotherapy outcomes (Alexander et al., 2017; Gopalakrishnan et al., 2018). Gut microbial enzymes like β-glucuronidase from *Clostridium* and *Escherichia* species can reactivate irinotecan metabolites in the gut, leading to severe diarrhea and gastrointestinal toxicity (Wallace et al., 2010). Selective enzyme inhibitors (e.g., DRB 156) are under clinical evaluation to mitigate these adverse effects (Stringer et al., 2009). Furthermore, bacterial thioguanine methyltransferase contributes to chemoresistance by converting thiopurine drugs into inactive metabolites (Huang et al., 2018).

In the context of immune checkpoint blockade therapy, high microbial diversity and the abundance of Akkermansia muciniphila, Ruminococcus bromii, and Bifidobacterium longum are associated with improved responses to PD-1/PD-L1 inhibitors across various cancers (Gopalakrishnan et al., 2018; Routy et al., 2018). Conversely, antibiotic use around treatment initiation reduces efficacy by disrupting gut microbiome balance and modulating immune cell function (Derosa et al., 2018). Fecal microbiota transplantation from responders shows promise in restoring immunotherapy sensitivity (Baruch et al., 2021).

Radiotherapy can damage the gut microbiota, leading to radiation proctitis and enteritis. Butyrate-producing bacteria like Eubacterium hallii and Roseburia intestinalis can ameliorate these toxicities by enhancing epithelial repair through IL-22/STAT3 pathways (Houlden et al., 2015). Trials such as RADIOTIDE CRC are investigating short-chain fatty acid supplementation to prevent radiation-induced gut damage (NCT05987121).

Perioperative antibiotic prophylaxis reduces postoperative infections but may increase cancer recurrence by depleting beneficial gut microbes, underscoring the importance of microbiome-sparing protocols (Mik et al., 2016) and precision microbiome approaches guided by recent multi-omics and therapeutic insights (Methé et al., 2012; Bindels et al., 2025; Liang et al., 2024). Engineered bacterial and probiotic therapies, including strains secreting anti–PD-L1 nanobodies, have demonstrated improved antitumor responses and immunotherapy outcomes (Gurbatri et al., 2022; Gopalakrishnan et al., 2018), allowing for personalized supportive care interventions (Dohlman et al., 2021; Alexander et al., 2017).

### 5.1 Diet, medications, lifestyle, and environmental modulators of the oncogenic microbiome

**FIGURE 2:**
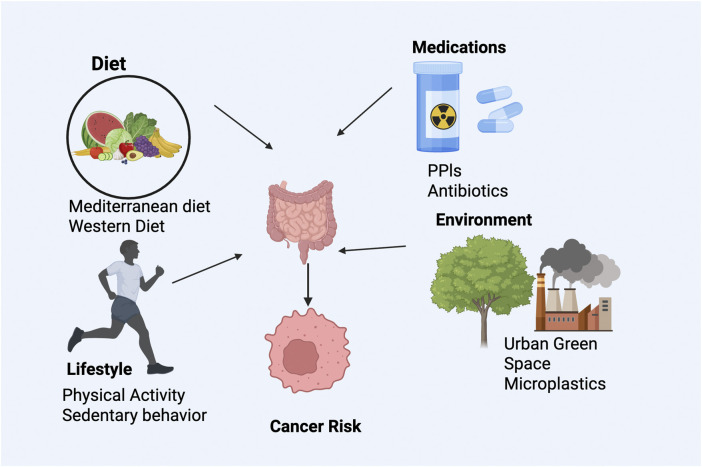
Modifiable factors influencing gut microbiome and cancer risk. Diet, medications, lifestyle, and environmental exposures shape microbial composition and function. Protective factors include fiber-rich diets and exercise, while Western diets, antibiotics, and pollutants promote dysbiosis, altering immune and metabolic pathways linked to GI cancer.

The gut microbiome’s composition and function are dynamically responsive to dietary, medicinal, lifestyle, and environmental influences, each profoundly affecting cancer risk and progression (Zitvogel et al., 2018; Turnbaugh et al., 2007). Dietary patterns significantly shape microbiota composition and metabolite production. For instance, fiber-rich Mediterranean diets have been consistently linked to increased populations of beneficial microbes, such as Roseburia and Faecalibacterium species, which produce protective short-chain fatty acids and reduce carcinogenic metabolites (David et al., 2014). Conversely, Western diets characterized by high intake of red meat, processed foods, and emulsifiers favor pathogenic microbial blooms, notably sulfate-reducing Bilophila wadsworthia, promoting pro-carcinogenic environments through increased production of DNA-damaging agents like hydrogen sulfide (Devkota et al., 2012) (Figure 2).

Medication use also profoundly influences microbiome dynamics. Proton pump inhibitors (PPIs) significantly alter microbial diversity by reducing gastric acidity, facilitating colonization of oral microbes in the gut and increasing colorectal cancer risk (Imhann et al., 2016). Broad-spectrum antibiotics cause lasting reductions in microbial diversity, significantly impairing immune checkpoint inhibitor efficacy (Gopalakrishnan et al., 2018). Conversely, non-steroidal anti-inflammatory drugs (NSAIDs) exhibit chemopreventive effects partly attributable to microbiome modulation via arachidonic acid pathways (Alexander et al., 2017).

Lifestyle factors such as physical activity and environmental exposures further modulate microbiome composition. Regular moderate-intensity exercise enhances the abundance of beneficial microbial taxa like Akkermansia and Eubacterium through improved gut motility and mucin turnover (Clarke et al., 2014). Sedentary behavior reduces these protective microbiota populations, promoting a pro-inflammatory state (Allen et al., 2018). Environmental factors, including exposure to urban green spaces, have been associated with increased microbial diversity and anti-inflammatory metabolite production, potentially reducing cancer risk (Ruokolainen et al., 2015). However, emerging evidence suggests adverse impacts of microplastic ingestion and heavy metal exposure on microbial community stability and redox balance, underscoring the complexity of environmental influences on the microbiome and their potential implications for cancer risk and prevention strategies (Leslie et al., 2022) (Table 1).

**TABLE 1:** Microbiome-targeted therapeutic strategies in gastrointestinal cancers. Summary of key microbiome-based interventions, main findings, and current development stages.

Strategy	Key findings	Development stage
Faecal microbiota transplantation (FMT)	Oral-capsule FMT accelerates microbiome recovery after HSCT and cuts grade ≥2 GVHD by 40% (Khoruts et al., 2021)	Phase III
Responder-FMT	Restores ICI benefit in 30%–50% of refractory melanoma/GI-cancer patients (Davar et al., 2021)	Phase II
Antibiotic-conditioned FMT	18% partial responses in heavily-pretreated GI cancers (Baruch et al., 2021)	Phase I/II
Engineered probiotics	*E. coli* Nissle secreting anti-PD-L1 nanobodies shrinks CRC in mice (Gurbatri et al., 2024)	Phase I
Prebiotic/synbiotic fibres	Resistant-starch supplementation restores butyrate and reduces adenoma multiplicity (O’Keefe et al., 2015)	Translational
Bacteriophage therapy	CRISPR-phage targeting *F. nucleatum* reduces tumour burden in *Apc*Min/ + mice (Bullman et al., 2017)	Pre-clinical

## 6 Cross-cutting challenges, knowledge gaps, and methodological priorities

Despite significant advances, translating microbiome science into routine gastro-oncology practice presents considerable challenges (Supplementary Figure S1). Establishing causality remains a key obstacle. While animal models have demonstrated that specific microbes like F. nucleatum and pks + *E. coli* can drive tumorigenesis, human studies remain largely correlative (Kostic et al., 2013; Louis et al., 2014). Innovative approaches, including Mendelian randomization using host microbiome-related SNPs, are promising but often hampered by population stratification and limited sample sizes (Wang et al., 2019). Large-scale bidirectional studies and causal mediation analyses are needed to clarify these complex interactions and provide robust evidence.

Standardization and reproducibility pose additional hurdles. Methodological variations in sample collection, DNA extraction, and bioinformatics pipelines hinder cross-study comparability (Costea et al., 2017). Initiatives like the Microbiome Quality Control Consortium have recommended standardized protocols, including mock community controls and comprehensive metadata reporting, to enable consistent and reproducible data integration (Sinha et al., 2017).

Spatial and functional resolution of microbiome data is another area requiring development. While bulk sequencing has illuminated general dysbiosis patterns, spatial transcriptomics and microbial FISH are now revealing micro-niches of bacteria within tumor microenvironments that could influence cancer behavior (Geller et al., 2017). Integrating spatial metabolomics with isotope tracing will deepen our understanding of local microbe-host interactions and nutrient exchanges (Bouslimani et al., 2016).

Regulatory science for live biotherapeutics is also critical. Current FDA guidelines treat engineered probiotics as biologics, requiring rigorous strain-level genomic characterization, stability assessments, and standardized potency assays (Yadav and Chauhan, 2021). Establishing consistent regulatory frameworks will be essential to facilitate safe and effective clinical translation of microbiome-based therapies.

Finally, equity and representation must be prioritized. Most metagenomic studies have been conducted in Western populations, limiting generalizability to other regions (Huttenhower et al., 2012). Global initiatives, including harmonized stool banks and open-access biobanks, are crucial for ensuring that the benefits of microbiome-based oncology reach all populations (Sinha et al., 2017).

The gut microbiome has emerged as a critical player in the pathogenesis and treatment of GI cancers, offering opportunities for both prevention and therapy. Dysbiosis drives oncogenesis through genotoxic, metabolic, epigenetic, and immunomodulatory pathways, while also influencing patient responses to chemotherapy, immunotherapy, and radiotherapy (Kostic et al., 2013; Tilg et al., 2018). This intricate interplay positions the microbiome as both a biomarker and a therapeutic target (Gopalakrishnan et al., 2018).

By 2030, we anticipate major milestones: the integration of multi-omics microbiome profiles into routine cancer screening, regulatory approval of microbiome-informed diagnostics, and widespread use of live biotherapeutics in clinical practice (Sampson et al., 2017). Advances in causal inference, standardized protocols, and federated data-sharing will accelerate discovery, while ensuring equitable access to microbiome-based interventions will be essential for addressing global disparities (Human Microbiome Project Consortium, 2012; Almeida et al., 2024).

In summary, the microbiome is at the nexus of host genetics, lifestyle, environment, and therapy response. Achieving precision onco-microbiomics requires collaborative efforts across disciplines, bridging basic research and clinical translation (Marshall and Warren, 1984). This approach holds the promise of transforming GI cancer care from reactive to proactive management, ultimately improving survival rates and patient quality of life worldwide (Qin et al., 2010).

## Supplementary material

SUPPLEMENTARY FIGURE 1 Cross-cutting challenges and priorities in microbiome–oncology. Key challenges include establishing causality, standardizing methodologies, and improving spatial/functional resolution. Priorities include regulatory frameworks for live biotherapeutics and ensuring global equity through diverse, standardized datasets.
