# Supplementary figures and images for: The gut microbiome and gastrointestinal cancers: mechanisms, biomarkers and therapeutic opportunities
Source: Front Physiol. 2025 Oct 30;16:1676796. doi: 10.3389/fphys.2025.1676796 (PMC12611654; doi:10.3389/fphys.2025.1676796)

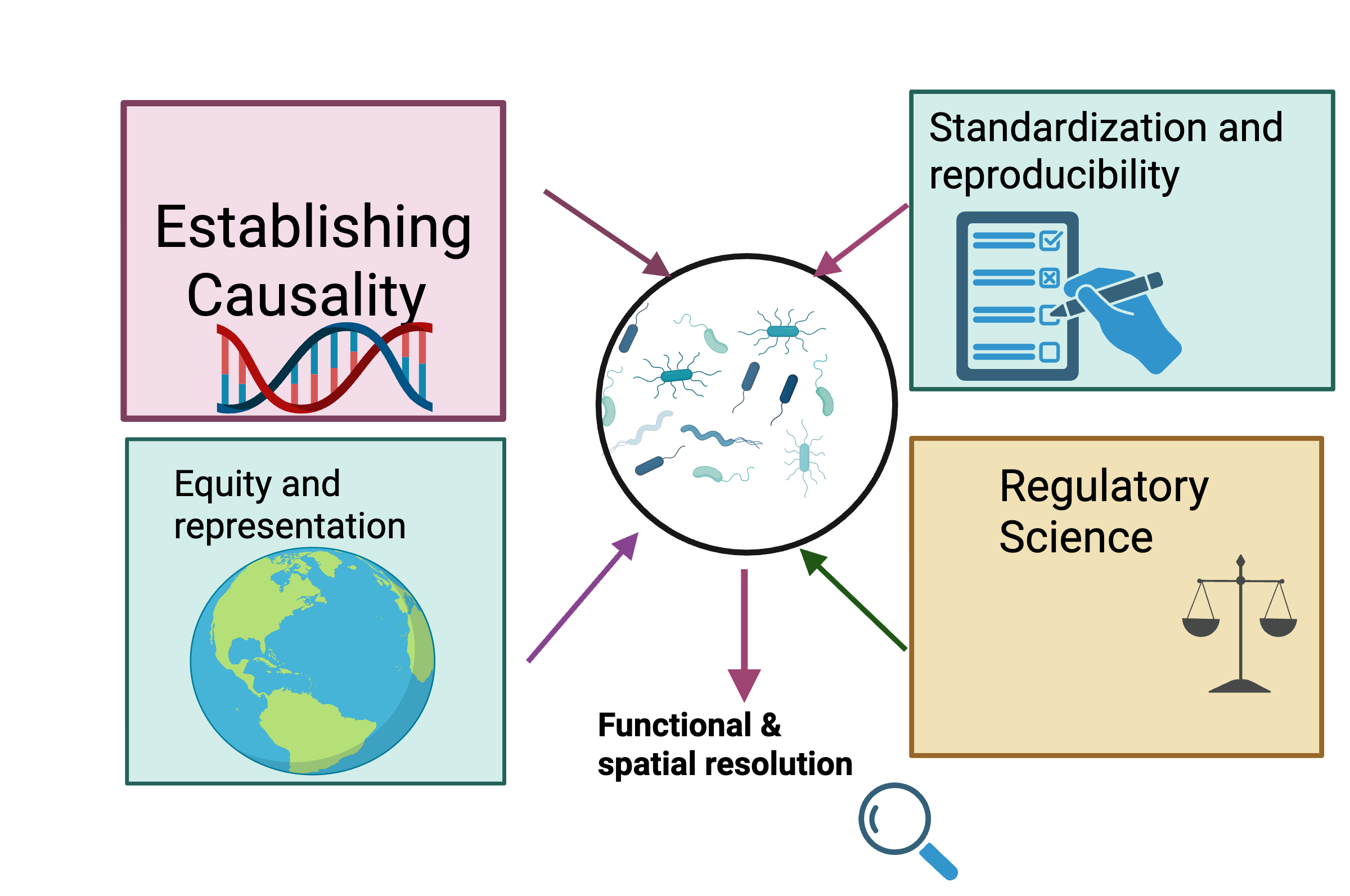

Supplement: Supplementary file 1 [file Image1.jpeg]
